# Risks of alcohol and drug use disorders in prostate cancer survivors: a national cohort study

**DOI:** 10.1093/jncics/pkad046

**Published:** 2023-06-30

**Authors:** Casey Crump, Pär Stattin, James D Brooks, Jan Sundquist, Alexis C Edwards, Weiva Sieh, Kristina Sundquist

**Affiliations:** Departments of Family Medicine and Community Health and of Population Health Science and Policy, Icahn School of Medicine at Mount Sinai, New York, NY, USA; Department of Surgical Sciences, Uppsala University, Uppsala, Sweden; Department of Urology, Stanford University School of Medicine, Stanford, CA, USA; Departments of Family Medicine and Community Health and of Population Health Science and Policy, Icahn School of Medicine at Mount Sinai, New York, NY, USA; Center for Primary Health Care Research, Department of Clinical Sciences, Lund University, Malmö, Sweden; Department of Psychiatry, Virginia Commonwealth University, Richmond, VA, USA; Department of Epidemiology, University of Texas MD Anderson Cancer Center, Houston, TX, USA; Departments of Family Medicine and Community Health and of Population Health Science and Policy, Icahn School of Medicine at Mount Sinai, New York, NY, USA; Center for Primary Health Care Research, Department of Clinical Sciences, Lund University, Malmö, Sweden

## Abstract

**Background:**

Prostate cancer (PC) survivors may potentially use substances to cope with psychological distress or poorly controlled physical symptoms. Little is known, however, about the long-term risks of alcohol use disorder (AUD) or drug use disorders in men with PC.

**Methods:**

A national cohort study was conducted in Sweden of 180 189 men diagnosed with PC between 1998 and 2017 and 1 801 890 age-matched population-based control men. AUD and drug use disorders were ascertained from nationwide records through 2018. Cox regression was used to compute hazard ratios (HRs) while adjusting for sociodemographic factors and prior psychiatric disorders. Subanalyses examined differences by PC treatment from 2005 to 2017.

**Results:**

Men with high-risk PC had increased risks of both AUD (adjusted HR = 1.44, 95% confidence interval [CI] = 1.33 to 1.57) and drug use disorders (adjusted HR = 1.93, 95% CI = 1.67 to 2.24). Their AUD risk was highest in the first year and was no longer significantly elevated 5 years after PC diagnosis, whereas their drug use disorders risk remained elevated 10 years after PC diagnosis (adjusted HR = 2.26, 95% CI = 1.45 to 3.52), particularly opioid use disorder (adjusted HR = 3.07, 95% CI = 1.61 to 5.84). Those treated only with androgen-deprivation therapy had the highest risks of AUD (adjusted HR = 1.91, 95% CI = 1.62 to 2.25) and drug use disorders (adjusted HR = 2.23, 95% CI = 1.70 to 2.92). Low- or intermediate-risk PC was associated with modestly increased risks of AUD (adjusted HR = 1.38, 95% CI = 1.30 to 1.46) and drug use disorders (adjusted HR = 1.19, 95% CI = 1.06 to 1.34).

**Conclusions:**

In this large cohort, men with PC had significantly increased risks of both AUD and drug use disorders, especially those with high-risk PC and treated only with androgen-deprivation therapy. PC survivors need long-term psychosocial support and timely detection and treatment of AUD and drug use disorders.

Prostate cancer (PC) is the most commonly diagnosed cancer among men in the United States, Europe, and many countries worldwide (1,2). More than 3.6 million men currently living in the United States have been diagnosed with PC (nearly 5 times more than any other cancers), and this number is expected to increase to more than 5 million by 2030 (3). PC survivors may use substances in an attempt to cope with psychological distress or poorly controlled physical symptoms (4). Such use could potentially lead to substance use disorders (SUDs), which may worsen quality of life and health outcomes (5,6). A better understanding of SUD risks is needed to inform psychosocial support interventions and improve outcomes in the growing number of PC survivors.

Most studies of substance use in relation to cancer have focused on alcohol or drugs as potential risk factors for cancer incidence (7-9). Less is known, however, about SUDs as potential outcomes in cancer survivors. A systematic review of 21 studies of patients with cancer (the most common sites were breast or head and neck) found that SUD rates varied widely across studies (2% (10) to 35% (11)), with a median rate of 18% for opioids and 25% for alcohol (4). Most studies, however, have been limited by lack of information about cancer treatment, inability to assess confounding, or aggregating all substances (4). SUDs have rarely been examined in men with PC, and the few studies to do so have lacked a comparison group without PC (12,13). Aggressive PC has previously been associated with increased risks of depression and suicide, which persisted 10 years after PC diagnosis (14). However, no studies have examined SUDs in PC survivors with sufficient follow-up and sample sizes to estimate long-term (≥5-year) risks and to identify the most susceptible subgroups that may benefit most from psychosocial support and other preventive interventions. Large population-based cohorts are needed to enable well-powered assessment of long-term risks of specific SUDs, susceptible time periods, and high-risk subgroups.

We sought to address these knowledge gaps using nationwide data in Sweden. Our goals were to determine the long-term risks of alcohol use disorder (AUD) and drug use disorders for men with PC with different prognoses in a large population-based cohort and to identify periods of heightened risk after PC diagnosis. We hypothesized that men with high-risk PC have increased long-term risks of both AUD and drug use disorders.

## Methods

### Study population and PC ascertainment

In the National Prostate Cancer Register (NPCR) of Sweden, we identified 183 495 men who were diagnosed with PC between 1998 and 2017 (14). The NPCR captures 98% of all incident PC cases since 1998 compared with the Swedish National Cancer Register, to which reporting is mandated by law (15). The NPCR contains data on cancer characteristics, including tumor grade according to Gleason score, disease stage according to the TNM staging classification, and prostate-specific antigen (PSA) level at diagnosis (16-18). We excluded 3306 (2%) men who had missing data for any of these characteristics, leaving 180 189 (98%) men for analysis (14).

PC risk groups were defined at the time of diagnosis based on a modification used by NPCR of the National Comprehensive Cancer Network Practice Guidelines criteria (15,19). Low-risk PC was defined by clinical local stage T1 to T2, Gleason score 2 to 6, and PSA level under 10 ng/mL; intermediate-risk PC was defined by stage T1 to T2, with Gleason score 7 or PSA level 10 to 20 ng/mL. High-risk PC was defined by clinical stage T3 or T4, Gleason score of 8 or higher, or PSA of 20 ng/mL or higher at the time of diagnosis; it was further stratified as locally advanced (stage T3 and PSA 20 to <50 ng/mL), very advanced/regionally metastatic (stage T4 or N1 or PSA 50 to <100 ng/mL in the absence of distant metastases [M0 or Mx]), or distant metastases (stage M1 or PSA ≥100 ng/mL) (15,19). Primary treatment within 6 months after diagnosis also was identified from the NPCR. Androgen-deprivation therapy (ADT) was further identified using Anatomical Therapeutic Chemical codes L02AE (gonadotropin-releasing hormone [GnRH] analogues), L02BB (antiandrogens), and L02BX (other hormone antagonists) in the Swedish Prescribed Drug Register, which contains all medication prescriptions dispensed nationwide since July 1, 2005.

Each PC case was matched to 10 men randomly sampled from the general population who had the same birth year and month and were living in Sweden on the date of PC diagnosis for the respective case (ie, index date) (14). This study was approved by the Regional Ethical Review Board in Lund, Sweden. Participant consent was not required because this study used only pseudonymized registry-based secondary data.

### AUD and drug use disorders ascertainment

The primary outcomes were the earliest diagnosis of (1) AUD or (2) drug use disorders, which were ascertained from the index date (respective case’s PC diagnosis date) through December 31, 2018, and were examined separately. To enable more complete ascertainment, AUD and drug use disorders were identified using multiple sources, including *International Classification of Diseases, Tenth Revision (ICD-10)* codes in the Swedish In-Patient and Out-Patient registers and primary care records; alcohol- or drug-related offenses in the Swedish Suspicion and Crime register; and medications used for AUD treatment in the Swedish Prescribed Drug Register (see Supplementary Methods, available online). The In-Patient Register contains all primary and secondary hospital discharge diagnoses, with 86% coverage of the Swedish population starting in 1973 and 100% coverage since 1987 (20). The Swedish Out-Patient Register contains all diagnoses from specialty clinics nationwide starting in 2001. Primary care diagnoses previously collected by our group (21) were available for 20% of the Swedish population starting in 1998, 45% starting in 2001, and 90% starting in 2008 and onward. The Swedish Crime and Suspicion Registers contain nationwide records of all criminal convictions since 1973 and all suspected crimes since 1998.

Diagnostic codes for drug use disorders specifically indicate “mental and behavioral disorders due to psychoactive substance use,” which may include opioids, sedatives/hypnotics, or nonprescription and illicit substances (eg, cannabinoids, cocaine, hallucinogens). Therefore, a patient with cancer who is prescribed opioids or sedatives/hypnotics as part of their clinical care is not counted as having a drug use disorder unless a diagnosis is also registered for a mental or behavioral disorder resulting from use of these substances.

### Covariates

Other characteristics that may be associated with PC and AUD or drug use disorders were identified using Swedish national census and health registry data. Covariates included birth date (continuous and categorical by decade); birth country (Sweden/other); marital status (married/not married); education level (≤9, 10-12, >12 years); income (quartiles); region (large cities, other/Southern, other/Northern, unknown); and prior history of major depression, anxiety disorder, bipolar disorder, schizophrenia, AUD, or drug use disorders (each ascertained from 1973 up to the index date and modeled as a separate covariate). Psychiatric disorders were ascertained from the Swedish In-Patient and Out-Patient registers and primary care records using *ICD-10* codes (Supplementary Methods, available online). All covariates were more than 96% complete. Missing data were modeled as a separate category and had little effect on risk estimates because of their rarity.

### Statistical analysis

Cox regression was used to compute hazard ratios (HRs) and 95% confidence intervals (CIs) for AUD or drug use disorders in men with PC compared with matched controls, while adjusting for prior AUD or drug use disorders before the index date and other covariates and stratifying on matched sets. In a secondary analysis, we explored the association between PC and “new-onset AUD” after excluding 7418 (4%) PC cases and 94 791 (5%) controls who had a prior registration of AUD before the index date or “new-onset drug use disorder” after excluding 920 (0.5%) PC cases and 13 623 (0.8%) controls who had a prior registration of drug use disorder before the index date. The proportional hazards assumption was evaluated by examining log-log survival plots and was satisfied in each model.

To assess periods of susceptibility, the outcomes were assessed within specific time intervals after PC diagnosis (<3, 3 to <12 months; 1 to <2, 2 to <5, 5 to <10, ≥10 years) in separate models. The outcomes were further stratified by primary treatment modality (ADT only; radical radiation therapy with or without adjuvant ADT; radical prostatectomy; or radical prostatectomy followed by radiation therapy) using treatment data from 2005 to 2017 compared with controls. ADT was further examined as GnRH analogues vs antiandrogen monotherapy. The most common specific drug use disorders (opioid and sedative/hypnotic use disorders) were also examined in separate subanalyses.

In exploratory analyses, age-specific differences were assessed by stratifying by age at the index date (<55, 55-64, 65-74, 75-84, ≥85 years) while adjusting for age as a continuous variable within each stratum. To assess for temporal changes, we also explored associations between high-risk PC and AUD or drug use disorders after stratifying on calendar year of PC diagnosis (1998-2004, 2005-2009, 2010-2017). All statistical tests were 2-sided and used a significance level of .05. All analyses were conducted using Stata, version 15.1 (StataCorp LP, College Station, TX, USA).

## Results

Among 180 189 men with PC, 56% had low- or intermediate-risk PC and 44% had high-risk PC (14). Men with low- or intermediate-risk PC were diagnosed at a median (IQR) age of 67 (62-73) years and had a median (IQR) follow-up time of 7 (4-11) years. Men with high-risk PC were diagnosed at a median (IQR) age of 75 (68-81) years and had a median (IQR) follow-up time of 4 (2-8) years. Men in the control group had a median (IQR) follow-up time of 5 (2-9) years.

In 8.0 million person-years of follow-up, a total of 74 092 (4%) and 10 819 (0.5%) men (with or without PC) were identified with AUD or drug use disorders, respectively. In total, 13% of AUD cases and 11% of drug use disorder cases were identified only from the Swedish Suspicion and Crime registers and were not identifiable using only health care registries. At 5 years of follow-up, the cumulative incidences of AUD and drug use disorders, respectively, were 3% and 0.4% among men with PC and 1% and 0.2% among men in the control group. The median ages at registration of AUD or drug use disorders, respectively, were 68 and 70 years in men with low- or intermediate-risk PC, 75 and 76 years in men with high-risk PC, and 70 and 71 years in controls. Most drug use disorder diagnoses were either opioid (39%) or sedative/hypnotic (31%) use disorders, whereas the remainder were various others, including use of nonprescription substances.

Table 1 shows characteristics of men with PC, men in the control group, and all men with AUD or drug use disorders. Men with PC were more likely than controls to be Swedish born or married. Men with high-risk PC had lower education and income levels than controls, whereas men with low- or intermediate-risk PC had higher education or income levels. Men with AUD or drug use disorders were younger and more likely to be unmarried, have low income, live in large cities, or have a prior diagnosis of major depression or anxiety disorder.

**Table 1.** Characteristics of men with prostate cancer and men in the control group, 1998-2018, Sweden

	High-risk PC^a^	Low- or intermediate-risk PC^b^	Controls	AUD	Drug use disorders
	n = 78 951	n = 101 238	n = 1 801 890	n = 74 092 (3.7%)	n = 10 819 (0.5%)
	No. (%)	No. (%)	No. (%)	No. (%)	No. (%)
**Age at index date, y**					
<55	1372 (1.7)	5728 (5.7)	71 000 (3.9)	4242 (5.7)	1257 (11.6)
55-64	10 791 (13.7)	32 406 (32.0)	431 970 (24.0)	26 629 (35.9)	3861 (35.7)
65-74	27 258 (34.5)	45 054 (44.5)	723 120 (40.1)	32 764 (44.2)	3806 (35.2)
75-84	30 272 (38.3)	16 356 (16.2)	466 280 (25.9)	9672 (13.1)	1647 (15.2)
≥85	9258 (11.7)	1694 (1.7)	109 520 (6.1)	785 (1.1)	248 (2.3)
**Sweden born**	72 846 (92.3)	92 334 (91.2)	1 573 042 (87.3)	65 293 (88.1)	9174 (84.8)
**Marital status**					
Married	54 933 (69.6)	72 080 (71.2)	1 141 069 (63.3)	35 371 (47.7)	4651 (43.0)
Not married	24 018 (30.4)	29 157 (28.8)	606 285 (33.7)	38 718 (52.3)	6168 (57.0)
Unknown	0 (0.0)	1 (<0.1)	54 536 (3.0)	3 (<0.1)	0 (0.0)
**Education, y**					
≤9	37 659 (47.7)	32 615 (32.2)	748 594 (41.5)	29 030 (39.2)	4327 (40.0)
10-12	27 165 (34.4)	40 885 (40.4)	648 123 (36.0)	31 340 (42.3)	4700 (43.4)
>12	14 117 (17.9)	27 727 (27.4)	355 450 (19.7)	13 715 (18.5)	1791 (16.6)
Unknown	10 (<0.1)	11 (<0.1)	49 723 (2.8)	7 (<0.1)	1 (<0.1)
**Income, quartile**					
1st (highest)	15 832 (20.1)	39 920 (39.4)	468 355 (26.0)	17 406 (23.5)	1647 (15.2)
2nd	21 091 (26.7)	28 529 (28.2)	462 783 (25.7)	20 881 (28.2)	2555 (23.6)
3rd	22 291 (28.2)	20 252 (20.0)	438 894 (24.4)	22 831 (30.8)	3904 (36.1)
4th (lowest)	19 694 (24.9)	12 481 (12.3)	359 227 (19.9)	12 793 (17.3)	2662 (24.6)
Unknown	43 (0.1)	56 (0.1)	72 631 (4.0)	181 (0.2)	51 (0.5)
**Region**					
Large cities	34 100 (43.2)	53 073 (52.4)	825 039 (45.8)	41 476 (56.0)	6364 (58.8)
Other/Southern	29 884 (37.9)	33 257 (32.9)	618 625 (34.3)	22 093 (29.8)	3037 (28.1)
Other/Northern	14 957 (18.9)	14 899 (14.7)	310 444 (17.2)	10 512 (14.2)	1411 (13.0)
Unknown	10 (<0.1)	9 (<0.1)	47 782 (2.7)	11 (<0.1)	7 (0.1)
**Prior psychiatric disorders**					
Major depression	3167 (4.0)	5108 (5.1)	91 107 (5.1)	10 269 (13.9)	2728 (25.2)
Anxiety disorder	2814 (3.6)	4370 (4.3)	81 331 (4.5)	9715 (13.1)	2946 (27.2)
Bipolar disorder	346 (0.4)	626 (0.6)	11 708 (0.6)	1472 (2.0)	670 (6.2)
Schizophrenia	247 (0.3)	235 (0.2)	11 808 (0.7)	822 (1.1)	435 (4.0)
AUD	5774 (7.3)	7706 (7.6)	159 504 (8.9)	44 348 (59.9)	5054 (46.7)
Drug use disorders	432 (0.6)	665 (0.7)	16 260 (0.9)	4800 (6.5)	4047 (37.4)

a High-risk PC was defined by clinical stage T3-T4, Gleason score ≥8, or PSA ≥20 ng/mL at time of diagnosis. AUD = alcohol use disorder, PC = prostate cancer; PSA = prostate-specific antigen.

b Low-risk PC was defined by clinical stage T1-T2, Gleason score 2-6, and PSA <10 ng/mL and intermediate-risk PC by clinical stage T1-T2, with Gleason score 7 or PSA 10 to <20 ng/mL.

### PC and AUD risk

Men with high-risk PC had a more than 40% higher risk of AUD across the entire follow-up period compared with controls (adjusted HR = 1.44, 95% CI = 1.33 to 1.57) (Table 2). This risk was slightly higher in men with distant metastases (adjusted HR = 1.58, 95% CI = 1.26 to 1.97) than those with locally advanced disease (adjusted HR = 1.42, 95% CI = 1.29 to 1.57) or very advanced/regionally metastatic disease (adjusted HR = 1.45, 95% CI = 1.19 to 1.76) (Supplementary Table 1, available online). Risk of AUD was highest within the first year and was no longer significantly elevated at 5 years after PC diagnosis (Table 2). Low- or intermediate-risk PC was associated with a modestly increased risk of AUD (adjusted HR = 1.38, 95% CI = 1.30 to 1.46) that was limited to the first year after PC diagnosis.

**Table 2.** Associations between prostate cancer diagnosis (1998-2017) and risk of alcohol use disorder through 2018, Sweden

	AUD, No.		Adjusted HR (95% CI)^a^	*P*
	PC cases	Controls		
**High-risk PC**				
Entire follow-up period	1704	22 914	1.44 (1.33 to 1.57)	<.001
<3 mo	1152	13 957	1.51 (1.36 to 1.68)	<.001
3 to <12 mo	199	2661	1.64 (1.29 to 2.08)	<.001
1 to <2 y	112	2002	1.10 (0.83 to 1.46)	.67
2 to <5 y	176	2936	1.29 (1.03 to 1.61)	.03
5 to <10 y	65	1276	1.39 (0.92 to 2.10)	.12
≥10 y	0	82	—	—
**High-risk PC (2005-2017)^b^**				
ADT only	542	—	1.91 (1.62 to 2.25)	<.001
Radiation	330	—	1.25 (1.04 to 1.51)	.02
Radical prostatectomy	48	—	1.69 (1.05 to 2.74)	.03
Radical prostatectomy and radiation	30	—	1.17 (0.65 to 2.11)	.60
**Low- or intermediate-risk PC**				
Entire follow-up period	3477	44 843	1.38 (1.30 to 1.46)	<.001
<3 mo	2360	26 081	1.47 (1.37 to 1.57)	<.001
3 to <12 mo	453	5566	1.42 (1.20 to 1.68)	<.001
1 to <2 y	243	3949	1.17 (0.95 to 1.44)	.13
2 to <5 y	328	6209	1.13 (0.95 to 1.34)	.17
5 to <10 y	93	2765	1.05 (0.74 to 1.48)	.78
≥10 y	0	273	—	—
**Low- or intermediate-risk PC (2005-2017)^b^**				
Deferred treatment	1455	—	1.39 (1.27 to 1.51)	<.001
ADT only	273	—	1.62 (1.28 to 2.04)	<.001
Radiation	483	—	1.76 (1.50 to 2.05)	<.001
Radical prostatectomy	255	—	1.85 (1.52 to 2.26)	<.001
Radical prostatectomy and radiation	86	—	1.54 (1.07 to 2.21)	.02

a Adjusted for age, birth country, marital status, education, income, region, and prior history of psychiatric disorders (major depression, anxiety disorder, bipolar disorder, schizophrenia, AUD, drug use disorder) at index date. ADT = androgen-deprivation therapy; AUD = alcohol use disorder; CI = confidence interval; HR = hazard ratio; PC = prostate cancer.

b Subanalysis based on treatment data available between 2005 and 2017.

In men with high-risk PC, the risk of AUD varied significantly by PC treatment (*P *=* *.004) (Table 2). Those treated only with ADT (adjusted HR = 1.91, 95% CI = 1.62 to 2.25) had higher risks than those treated with radiation (adjusted HR = 1.25, 95% CI = 1.04 to 1.51) or radical prostatectomy (adjusted HR = 1.69, 95% CI = 1.05 to 2.74) (*P *=* *.001 and *P *=* *.64, respectively, for comparisons with ADT) (Table 2). AUD risk was significantly elevated among those treated with GnRH analogues (adjusted HR = 1.97, 95% CI = 1.64 to 2.38) or antiandrogen monotherapy (adjusted HR = 1.67, 95% CI = 1.15 to 2.42) (*P *=* *.43 for difference in hazard ratios). Very advanced/regionally metastatic PC and distant PC metastases were more common among all men treated only with ADT (17% and 16%, respectively) than among those treated with radiation (9% and 7%), radical prostatectomy (4% and 3%), or both radiation and radical prostatectomy (5% and 4%).

Among men with low- or intermediate-risk PC, the risk of AUD was significantly elevated regardless of treatment but with some heterogeneity (*P *=* *.008) (Table 2). AUD risk was slightly lower among those with primary deferred treatment (adjusted HR = 1.39, 95% CI = 1.27 to 1.51) than those treated with ADT only (adjusted HR = 1.62, 95% CI = 1.28 to 2.04), radiation (adjusted HR = 1.76, 95% CI = 1.50 to 2.05), or radical prostatectomy (adjusted HR = 1.85, 95% CI = 1.52 to 2.26) (*P *=* *.22, *P *=* *.009, and *P *=* *.009, respectively, for comparisons with no treatment).

High-risk and low- or intermediate-risk PC were associated with significantly increased risks of AUD in men either with or without a prior registration of AUD before the index date, but those with prior AUD had a moderately higher risk (*P *<* *.01 for difference in hazard ratios). In a secondary analysis, risk of “new-onset” AUD was assessed by excluding men with a prior registration of AUD (7418 [4%] PC cases and 94 791 [5%] controls), instead of adjusting for prior AUD in the entire group, as in the main analyses. Most risk estimates were moderately reduced, but the main findings remained statistically significant, including a 1.4-fold risk among men with high-risk PC in the first year after diagnosis (Supplementary Table 2, available online).

### PC and drug use disorders risk

**Figure 1.**
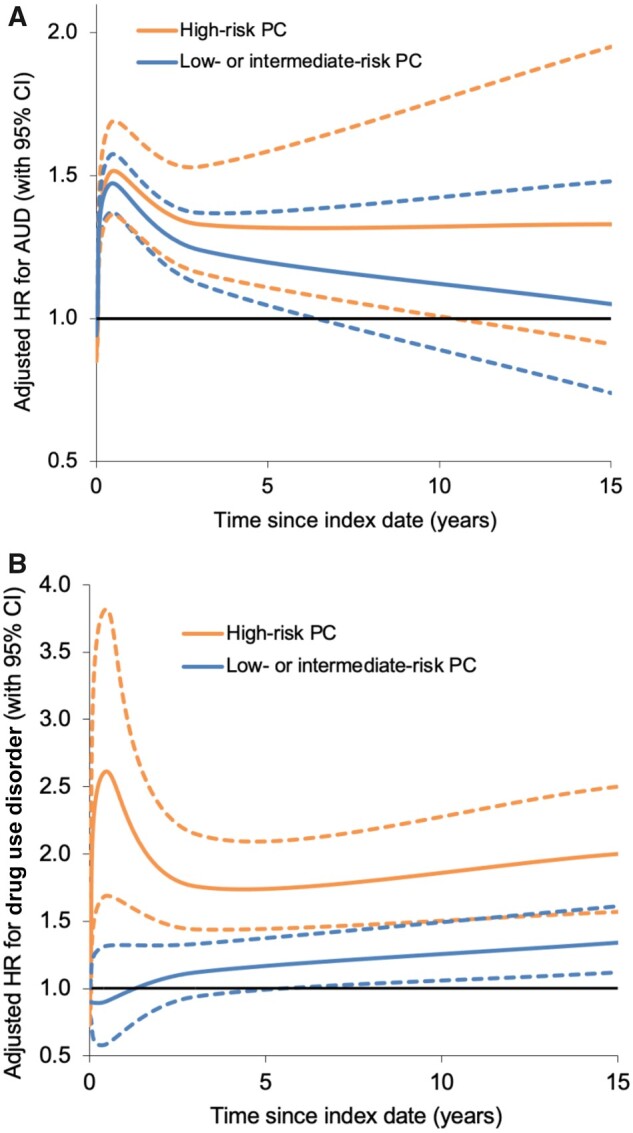
Adjusted hazard ratios for AUD (**A**) or drug use disorders (**B**) associated with high-risk PC or low- to intermediate-risk PC by time since index date, 1998-2018, Sweden (**dotted lines** represent 95% CI). AUD = alcohol use disorder; CI = confidence interval; HR = hazard ratio; PC = prostate cancer.

Men with high-risk PC had a nearly 2-fold risk of drug use disorders across the entire follow-up period compared with men in the control group (adjusted HR = 1.93, 95% CI = 1.67 to 2.24) (Table 3). This risk was higher in men with distant metastases (adjusted HR = 3.76, 95% CI = 2.63 to 5.36) than those with locally advanced disease (adjusted HR = 1.72, 95% CI = 1.45 to 2.05) or very advanced/regionally metastatic disease (adjusted HR = 1.63, 95% CI = 1.13 to 2.35) (Supplementary Table 3, available online). The relative rate of drug use disorders peaked in the first 3 months after high-risk PC diagnosis (adjusted HR = 3.72, 95% CI = 1.93 to 7.15) but was significantly elevated even at 10 years (adjusted HR = 2.26, 95% CI = 1.45 to 3.52) (Table 3). In contrast, low- or intermediate-risk PC was associated with only a modestly increased risk of drug use disorders across the entire follow-up period (adjusted HR = 1.19, 95% CI = 1.06 to 1.34) (Table 3). Figure 1 shows adjusted hazard ratios and 95% confidence intervals for AUD or drug use disorders by time since the index date, fitted using spline curves.

**Table 3.** Associations between prostate cancer diagnosis (1998-2017) and risk of any drug use disorder through 2018, Sweden

	Drug use disorders, No.		Adjusted HR (95% CI)^a^	*P*
	PC cases	Controls		
**High-risk PC**				
Entire follow-up period	404	3023	1.93 (1.67 to 2.24)	<.001
<3 mo	39	744	3.72 (1.93 to 7.15)	<.001
3 to <12 mo	55	428	1.54 (0.99 to 2.38)	.05
1 to <2 y	66	399	1.83 (1.24 to 2.70)	.002
2 to <5 y	109	736	1.89 (1.44 to 2.47)	<.001
5 to <10 y	94	528	1.92 (1.43 to 2.57)	<.001
≥10 y	41	188	2.26 (1.45 to 3.52)	<.001
**High-risk PC (2005-2017)^b^**				
ADT only	138	—	2.23 (1.70 to 2.92)	<.001
Radiation	76	—	1.80 (1.26 to 2.58)	.001
**Low- or intermediate-risk PC**				
Entire follow-up period	609	6620	1.19 (1.06 to 1.34)	.004
<3 mo	31	1348	0.97 (0.48 to 1.94)	.92
3 to <12 mo	84	892	0.93 (0.63 to 1.38)	.71
1 to <2 y	83	893	1.10 (0.78 to 1.55)	.60
2 to <5 y	167	1620	1.15 (0.92 to 1.43)	.23
5 to <10 y	163	1350	1.39 (1.11 to 1.73)	.004
≥10 y	81	517	1.26 (0.92 to 1.72)	.16
**Low- or intermediate-risk PC (2005-2017)^b^**				
Deferred treatment	246	—	1.11 (0.91 to 1.35)	.31
ADT only	61	—	2.30 (1.48 to 3.57)	<.001
Radiation	70	—	0.91 (0.63 to 1.32)	.63

a Adjusted for age, birth country, marital status, education, income, region, and prior history of psychiatric disorders (major depression, anxiety disorder, bipolar disorder, schizophrenia, AUD, drug use disorder) at index date. ADT = androgen-deprivation therapy; AUD = alcohol use disorder; CI = confidence interval; HR = hazard ratio; PC = prostate cancer.

b Subanalysis based on treatment data available between 2005 and 2017.

Among men with high-risk PC, the risk of drug use disorders was significantly elevated in those treated with ADT only (adjusted HR = 2.23, 95% CI = 1.70 to 2.92) or with radiation (adjusted HR = 1.80, 95% CI = 1.26 to 2.58) (*P *=* *.36 for difference in hazard ratios) (Table 3). Drug use disorders risk was significantly increased in men treated with GnRH analogues (adjusted HR = 2.44, 95% CI = 1.82 to 3.27) but not with antiandrogen monotherapy (adjusted HR = 1.28, 95% CI = 0.60 to 2.70). All other treatment categories had too few drug use disorder cases for meaningful analysis. Men with low- or intermediate-risk PC treated only with ADT also had a significantly increased risk of drug use disorders (adjusted HR = 2.30, 95% CI = 1.48 to 3.57), particularly those treated with GnRH analogues (adjusted HR = 2.66, 95% CI = 1.53 to 4.65).

Associations between high-risk or low- or intermediate-risk PC and drug use disorders did not vary significantly by whether there was a prior registration of drug use disorder before the index date (*P *>* *.05 for differences in hazard ratios). In a secondary analysis, new-onset drug use disorders were assessed by excluding men with a prior registration of drug use disorder (920 [0.5%] PC cases and 13 623 [0.8%] controls) as an alternative to adjusting for prior drug use disorder, as in the main analyses. Most risk estimates were little changed (Supplementary Table 4, available online). Across the entire follow-up period, the adjusted hazard ratio for new-onset drug use disorders in men with high-risk PC was 1.86 (95% CI = 1.60 to 2.15).

In analyses of specific drug use disorders, men with high-risk PC had an increased risk of opioid use disorder (OUD) across the entire follow-up period (adjusted HR = 1.69, 95% CI = 1.39 to 2.06) and especially at 5 to 10 years (adjusted HR = 2.03, 95% CI = 1.39 to 2.97) and 10 years and beyond (adjusted HR = 3.07, 95% CI = 1.61 to 5.84) after PC diagnosis (Supplementary Table 5, available online). They also had an increased risk of sedative/hypnotic use disorders in the first 3 months after PC diagnosis (adjusted HR = 3.19, 95% CI = 1.24 to 8.22) but not across the entire follow-up period (adjusted HR = 1.20, 95% CI = 0.90 to 1.59), except among those treated only with ADT (adjusted HR = 1.95, 95% CI = 1.14 to 3.33) (Supplementary Table 6, available online).

### Other secondary analyses

**Figure 2.**
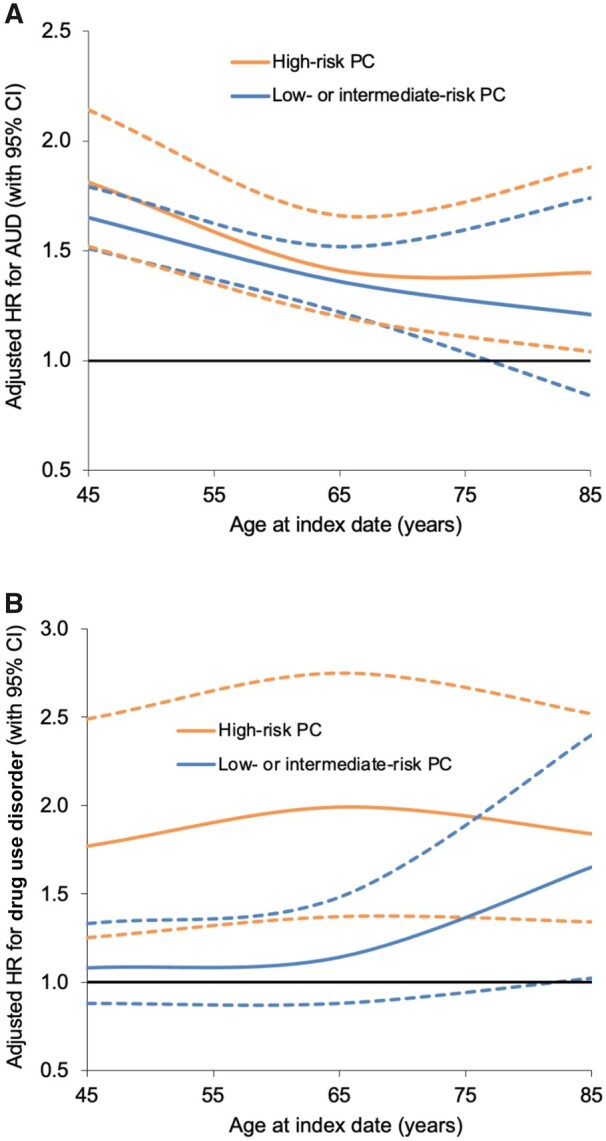
Adjusted hazard ratios for AUD (**A**) or drug use disorders (**B**) associated with high-risk PC or low- to intermediate-risk PC by age at index date, 1998-2018, Sweden (**dotted lines** represent 95% CI). AUD = alcohol use disorder; CI = confidence interval; HR = hazard ratio; PC = prostate cancer.

Stratifying on age at PC diagnosis, risks of either AUD or drug use disorders had significant heterogeneity (*P *<* *.001). For low- or intermediate-risk and high-risk PC, the relative rate for AUD was highest in men diagnosed with PC younger than 55 years of age (Supplementary Table 7, available online). In contrast, the relative rate of drug use disorders was highest in men diagnosed with low- or intermediate-risk PC at older ages (≥75 years) and was consistently elevated across all men 65 years of age or older with high-risk PC (Supplementary Table 8, available online). Figure 2 shows adjusted hazard ratios for AUD or drug use disorders associated with high-risk PC by age at the index date.

Stratifying by year of PC diagnosis, no consistent temporal patterns were found. The risk of AUD appeared to peak among men diagnosed with high-risk PC in 2005 to 2009 (adjusted HR = 1.81, 95% CI = 1.55 to 2.11), whereas drug use disorders risk peaked among those diagnosed between 1998 and 2004 (adjusted HR = 2.32, 95% CI = 1.37 to 3.92) or between 2010 and 2017 (adjusted HR = 2.27, 95% CI = 1.56 to 3.31) (Supplementary Table 9, available online).

## Discussion

In this large, population-based cohort, men diagnosed with high-risk PC had more than a 40% subsequent increased risk of AUD and 90% increased risk of drug use disorders compared with men in the control group without PC, after adjusting for sociodemographic factors and prior psychiatric diagnoses. Their risk of AUD was highest in the first year and was no longer significantly elevated at 5 years after PC diagnosis, whereas their risk of drug use disorders (particularly OUD) remained elevated 10 years after PC diagnosis. Men treated with ADT had the highest risks of both AUD and drug use disorders. Men with low- or intermediate-risk PC had only modestly increased risks of AUD and drug use disorders.

To our knowledge, this study is the first to examine long-term (≥5-year) risks of AUD and drug use disorders associated with PC in a large, population-based cohort. A previous study of 14 277 US Medicare patients 66 years of age or older with advanced-stage PC showed an 11% overall prevalence of SUDs but did not include a comparison group without PC (12). We previously reported that men with high-risk PC had approximately an 80% increased risk of major depression and more than 2-fold risk of death by suicide compared with same-aged men without PC and that these risks remained elevated 10 years after PC diagnosis (14). Other studies also have reported a high prevalence of psychosocial distress among patients with PC but without assessing SUDs. A meta-analysis of 27 studies with a pooled sample size of 4494 men with PC, for example, reported a 15% to 18% prevalence of clinically significant depression (22). A Surveillance, Epidemiology, and End Results–Medicare study of 50 856 men with localized PC and 2 to 7 years of follow-up reported that 20% developed mental illness (a composite of depression, anxiety, and suicide) (23).

The present study extends prior evidence by examining long-term risks of specific SUDs and periods of heightened risk in different PC risk groups and by PC treatment. Men with high-risk PC had substantially increased risks of AUD that were highest in the first year and even higher relative rates of drug use disorders than men in the control group, especially sedatives/hypnotics (3-fold) at less than 3 months and opioids (3-fold) at 10 years or more after PC diagnosis. Moreover, those treated only with ADT had the highest risks of AUD (1.9-fold) and drug use disorders (2.2-fold) and specifically risks of sedative/hypnotic use disorder (1.9-fold) and OUD (2.2-fold). These findings were consistent with elevated risks of major depression and suicide that we previously reported among men treated with ADT (14) and with a prior meta-analysis that reported an association between ADT use and depression, even in men with localized disease (24). The higher risks observed in men who received noncurative ADT may be related to side effects of ADT (25) as well as more aggressive cancers in such men with ensuing poorer prognosis and less hope of cure than men treated with curatively intended therapy, such as radical radiation therapy or radical prostatectomy. In this cohort, men treated only with ADT were more likely to have very advanced or metastatic disease than those treated with radiation or radical prostatectomy. Despite elevated relative risks in particular among men with advanced PC and men treated with ADT, however, it should be noted that the cumulative incidences of AUD and drug use disorders were modest both in men with PC and in men in the control group.

PC survivors may potentially use substances in an attempt to cope with psychological distress or poorly controlled physical symptoms (22,26-28). The stronger associations for drug use disorders (including OUD and sedative/hypnotic use disorder) than for AUD suggest that these findings may be partly related to cancer pain management (4). These findings have important clinical implications because substance use may compromise cancer treatment adherence (29), symptom management (30), quality of life (31), and long-term health outcomes (12,13). AUD and drug use disorders have previously been associated with higher health care utilization, cost of care, and mortality in men with high-risk PC (12). Follow-up care for PC survivors should include psychosocial support, particularly shortly after PC diagnosis, to help reduce psychosocial distress and prevent the development of AUD or drug use disorders. We found no decrease in risk of AUD or drug use disorders in later calendar periods, which may suggest no major improvement in psychosocial support for men with PC. American Cancer Society guidelines currently recommend alcohol use counseling and screening at least annually for psychosocial distress and depression (but not specifically AUD and drug use disorders) in men with PC (32). The United States Preventive Services Task Force also recommends screening for AUD and drug use disorders in all adult primary care settings (33-35). Validated screening tools exist that can quickly and accurately identify AUD and drug use disorders in primary care (36-38). Psychosocial support and clinical screening for AUD and drug use disorders are warranted in PC survivors, especially men with high-risk disease and ADT use. Positive screens should be followed by a brief intervention focused on patient education and prompt referral for treatment (36-38), which may improve long-term health outcomes (12,13).

###  

A key strength of this study was its large, national cohort design, which provided the high statistical power needed to examine PC risk groups, narrowly defined periods of susceptibility, and potential heterogeneity of risk by PC treatment. The inclusion of AUD and drug use disorder diagnoses from primary care, where most cases are diagnosed (21), enabled more complete ascertainment than in prior studies and thus more valid risk estimates. The availability of alcohol- and drug-related offenses from the Swedish Suspicion and Crime registers also enabled more complete capture of cases that could not be identified using only health care records. We were able to assess long-term risks of both AUD and drug use disorders in a national population while controlling for multiple potential confounders. Previously reported incidences of AUD, drug use disorders, and other major mental health outcomes are comparable between Sweden and the United States (21,39,40).

This study also had certain limitations. AUD and drug use disorders were identified from *ICD-10* codes and other registry sources, but more detailed clinical data were unavailable for validation. Their validity, however, has previously been supported by their prevalence, sex ratio, sibling correlations, and associations with well-documented risk factors (21). It is possible that SUDs were more likely to be identified in men with PC because of greater contact with the health care system (ie, detection bias), particularly AUD in the first year after PC diagnosis. Certain alcohol- or drug-related offenses in the Swedish Suspicion and Crime registers could also represent risky substance use rather than a true SUD. Despite a large cohort that enabled key subgroup analyses, statistical power for certain subgroups was still limited. This study also was performed in Sweden and will need replication in more diverse populations to explore for racial/ethnic heterogeneity. In a previous US study, SUDs had higher prevalences in people from racial/ethnic minoritized groups with PC (13).

In this large population-based cohort, men with PC had increased risks of both AUD and drug use disorders, especially those with high-risk PC and ADT use. Among men with high-risk PC, the risk of drug use disorders (particularly OUD) remained elevated 10 years and longer after PC diagnosis. PC survivors need psychosocial support, particularly shortly after PC diagnosis, and long-term follow-up for prevention, timely detection, and treatment of AUD and drug use disorders.

## Supplementary Material

pkad046_Supplementary_Data

## Data Availability

The national registry data on which this study was based were analyzed under strict confidentiality agreements with Swedish authorities. Because of ethical concerns, the supporting data cannot be made openly available. Further information about the health registers is available from the Swedish National Board of Health and Welfare: https://www.socialstyrelsen.se/en/statistics-and-data/registers/.
