# Supplementary material for: Risks of alcohol and drug use disorders in prostate cancer survivors: a national cohort study
Source: JNCI Cancer Spectr. 2023 Jun 30;7(4):pkad046. doi: 10.1093/jncics/pkad046 (PMC10393870; doi:10.1093/jncics/pkad046)
Supplement: pkad046_Supplementary_Data [file pkad046_supplementary_data.pdf]

SUPPLEMENTARY MATERIAL

Contents

Supplementary Methods. Ascertainment of AUD, DUD, and psychiatric disorders.

Supplementary Table 1. Associations between high-risk PC (1998-2017), stratified by locally advanced, very advanced, or distant metastases, and risk of AUD through 2018, Sweden.

Supplementary Table 2. Associations between PC diagnosis (1998-2017) and risk of AUD through 2018, after excluding men with any diagnosis of AUD before index date.

Supplementary Table 3. Associations between high-risk PC (1998-2017), stratified by locally advanced, very advanced, or distant metastases, and risk of DUD through 2018, Sweden.

Supplementary Table 4. Associations between PC diagnosis (1998-2017) and risk of DUD through 2018, after excluding men with any diagnosis of DUD before index date.

Supplementary Table 5. Associations between PC diagnosis (1998-2017) and risk of opioid use disorder through 2018, Sweden.

Supplementary Table 6. Associations between PC diagnosis (1998-2017) and risk of sedative/hypnotic use disorder through 2018, Sweden.

Supplementary Table 7. Associations between PC diagnosis (1998-2017) and risk of AUD through 2018, stratified by age at index date.

Supplementary Table 8. Associations between PC diagnosis (1998-2017) and risk of DUD through 2018, stratified by age at index date.

Supplementary Table 9. Associations between high-risk PC (1998-2017), stratified by year of diagnosis, and risk of AUD or DUD within the next 2 years.

Supplementary Methods. Ascertainment of AUD, DUD, and psychiatric disorders.

Substance use and psychiatric disorders were identified using the following ICD codes:

Alcohol Use Disorder (AUD)

ICD-8: 291, 303, 357F (357.5), 425F (425.5), 535D (535.3), 571A-D (571.0-571.3), 980, V79B (V79.1)
ICD-9: 291, 303, 305A (305.0), 357F (357.5), 425F (425.5), 535D (535.3), 571A-D (571.0-571.3), 980, V79B (V79.1)
ICD-10: F10 (excluding F10.0), Z50.2, Z71.4, E24.4, G31.2, G62.1, G72.1, I42.6, K29.2, K70.0-K70.9, K85.2, K86.0, O35.4, T51.0-T51.9

AUD was further identified from the Suspicion and Crime Registers for individuals who had at least two convictions of drunk driving (law 1951:649) or drunk in charge of maritime vessel (law 1994:1009), and from the Prescribed Drug Register for those with a prescription for disulfiram (N07BB01), acamprosate (N07BB03), or naltrexone (N07BB04).

Drug Use Disorders (DUD)

ICD-8: 304
ICD-9: 292, 304, 305 (excluding 305.0 and 305.1)
ICD-10: F10-F19 (excluding F10 and F17)

DUD was further identified from the Suspicion Register by codes 3070, 5010, 5011, and 5012; from the Crime Register by references to laws covering narcotics (law 1968:64, paragraph 1, point 6) and drug-related driving offences (law 1951:649, paragraph 4, Subsection 2 and paragraph 4A, Subsection 2).

Psychiatric Disorders (modeled as covariates)

Major depression (ICD-10: F32-F33)
Anxiety disorders (ICD-10: F40-F42)
Bipolar disorder (ICD-10: F31)
Schizophrenia (ICD-10 F20)

Supplementary Table 1. Associations between high-risk PC (1998-2017), stratified by locally advanced, very advanced, or distant metastases, and risk of AUD through 2018, Sweden.

Time after PC diagnosis	AUD, n		Reduced model[a]	Full model[b]	
	PC cases	Controls	HR (95% CI)	HR (95% CI)	*P*
**High-risk PC/locally advanced**					
Entire follow-up period	1,193	15,367	1.40 (1.29, 1.51)	1.42 (1.29, 1.57)	<0.001
<3 months	793	9,228	1.43 (1.29, 1.58)	1.45 (1.28, 1.64)	<0.001
3 to <12 months	146	1,725	1.64 (1.30, 2.08)	1.73 (1.29, 2.32)	<0.001
1 to <2 years	79	1,342	1.23 (0.91, 1.64)	1.14 (0.80, 1.61)	0.46
2 to <5 years	126	2,056	1.21 (0.96, 1.54)	1.30 (1.00, 1.70)	0.05
5 to <10 years	49	950	1.33 (0.86, 2.06)	1.26 (0.78, 2.04)	0.34
≥10 years	0	66	--	--	--
**High-risk PC/very advanced**					
Entire follow-up period	301	4,225	1.63 (1.39, 1.91)	1.45 (1.19, 1.76)	<0.001
<3 months	214	2,593	1.90 (1.56, 2.31)	1.63 (1.27, 2.10)	<0.001
3 to <12 months	28	501	1.30 (0.82, 2.07)	1.23 (0.71, 2.14)	0.47
1 to <2 years	20	372	1.30 (0.75, 2.27)	0.94 (0.48, 1.87)	0.87
2 to <5 years	28	528	1.06 (0.64, 1.76)	1.14 (0.65, 1.97)	0.65
5 to <10 years	11	217	1.73 (0.71, 4.17)	2.17 (0.82, 5.76)	0.12
≥10 years	0	14	--	--	--
**High-risk PC/distant metastases**					
Entire follow-up period	210	3,322	1.55 (1.29, 1.87)	1.58 (1.26, 1.97)	<0.001
<3 months	145	2,136	1.70 (1.35, 2.14)	1.78 (1.33, 2.37)	<0.001
3 to <12 months	25	435	1.50 (0.90, 2.50)	1.83 (1.00, 3.35)	0.05
1 to <2 years	13	288	0.98 (0.53, 1.83)	1.06 (0.55, 2.06)	0.86
2 to <5 years	22	352	1.56 (0.88, 2.74)	1.29 (0.68, 2.45)	0.44
5 to <10 years	5	109	1.22 (0.36, 4.16)	1.29 (0.32, 5.17)	0.72
≥10 years	0	2	--	--	--

[a]Adjusted for age, birth country, marital status, education, income, and region.
[b]Additionally adjusted for prior history of psychiatric disorders (major depression, anxiety disorder, bipolar disorder, schizophrenia, AUD, DUD) at index date.
AUD = alcohol use disorder, CI = confidence interval, DUD = drug use disorders, HR = hazard ratio, PC = prostate cancer

Supplementary Table 2. Associations between PC diagnosis (1998-2017) and risk of AUD through 2018, after excluding men with any diagnosis of AUD before index date.

Time after PC diagnosis	AUD, n		Adjusted HR (95% CI)[a]	*P*
	PC cases	Controls		
**High-risk PC**				
Entire follow-up period	865	12,365	1.21 (1.10, 1.33)	<0.001
<3 months	505	6,264	1.38 (1.21, 1.56)	<0.001
3 to <12 months	108	1,559	1.40 (1.07, 1.84)	0.01
1 to <2 years	61	1,341	0.77 (0.56, 1.05)	0.09
2 to <5 years	133	2,121	0.99 (0.79, 1.24)	0.93
5 to <10 years	58	1,018	1.18 (0.80, 1.74)	0.41
≥10 years	0	62	--	--
**High-risk PC (2005-2017)[b]**				
ADT only	209	--	1.70 (1.38, 2.09)	<0.001
Radiation	175	--	1.22 (0.99, 1.51)	0.06
Radical prostatectomy	26	--	2.28 (1.28, 4.06)	0.005
Radical prostatectomy and radiation	21	--	1.14 (0.62, 2.08)	0.68
**Low- or intermediate-risk PC**				
Entire follow-up period	2,096	24,618	1.13 (1.06, 1.20)	<0.001
<3 months	1,303	11,876	1.30 (1.21, 1.41)	<0.001
3 to <12 months	290	3,225	1.16 (0.98, 1.38)	0.08
1 to <2 years	163	2,604	0.85 (0.69, 1.05)	0.14
2 to <5 years	265	4,512	0.78 (0.66, 0.92)	0.004
5 to <10 years	75	2,173	0.73 (0.52, 1.02)	0.06
≥10 years	0	228	--	--
**Low- or intermediate-risk PC (2005-2017)[b]**				
Deferred treatment	794	--	1.25 (1.13, 1.38)	<0.001
ADT only	132	--	1.49 (1.13, 1.95)	0.005
Radiation	287	--	1.57 (1.33, 1.86)	<0.001
Radical prostatectomy	184	--	1.48 (1.20, 1.82)	<0.001
Radical prostatectomy and radiation	45	--	1.29 (0.86, 1.93)	0.22

[a]Adjusted for age, birth country, marital status, education, income, region, and prior history of psychiatric disorders (major depression, anxiety disorder, bipolar disorder, schizophrenia, DUD) at index date.

AUD = alcohol use disorder, CI = confidence interval, DUD = drug use disorders, HR = hazard ratio, PC = prostate cancer

Supplementary Table 3. Associations between high-risk PC (1998-2017), stratified by locally advanced, very advanced, or distant metastases, and risk of DUD through 2018, Sweden.

Time after PC diagnosis	DUD, n		Reduced model[a]	Full model[b]	
	PC cases	Controls	HR (95% CI)	HR (95% CI)	*P*
**High-risk PC/locally advanced**					
Entire follow-up period	267	2,114	1.66 (1.42, 1.94)	1.72 (1.45, 2.05)	<0.001
<3 months	24	475	1.95 (1.10, 3.48)	4.09 (1.81, 9.27)	0.001
3 to <12 months	28	277	1.32 (0.83, 2.10)	1.34 (0.75, 2.40)	0.33
1 to <2 years	47	283	2.24 (1.53, 3.29)	2.22 (1.43, 3.45)	<0.001
2 to <5 years	72	529	1.59 (1.19, 2.13)	1.52 (1.11, 2.10)	0.01
5 to <10 years	61	404	1.34 (0.97, 1.85)	1.43 (1.02, 2.00)	0.04
≥10 years	35	146	2.10 (1.32, 3.34)	2.39 (1.48, 3.88)	<0.001
**High-risk PC/very advanced**					
Entire follow-up period	60	537	1.54 (1.11, 2.14)	1.63 (1.13, 2.35)	0.009
<3 months	6	146	1.44 (0.47, 4.39)	0.95 (0.19, 4.82)	0.95
3 to <12 months	11	90	1.39 (0.66, 2.92)	1.47 (0.62, 3.47)	0.39
1 to <2 years	10	69	1.07 (0.48, 2.38)	0.99 (0.41, 2.39)	0.99
2 to <5 years	15	117	1.66 (0.87, 3.16)	1.83 (0.94, 3.58)	0.08
5 to <10 years	16	86	3.05 (1.52, 6.13)	3.07 (1.44, 6.56)	0.004
≥10 years	2	29	0.67 (0.14, 3.32)	1.06 (0.19, 5.85)	0.95
**High-risk PC/distant metastases**					
Entire follow-up period	77	372	3.09 (2.24, 4.27)	3.76 (2.63, 5.36)	<0.001
<3 months	9	123	5.95 (2.17, 16.32)	8.06 (2.13, 30.42)	0.002
3 to <12 months	16	61	2.63 (1.30, 5.31)	3.58 (1.59, 8.08)	0.002
1 to <2 years	9	47	1.77 (0.71, 4.42)	1.83 (0.66, 5.04)	0.24
2 to <5 years	22	90	3.31 (1.82, 6.02)	4.22 (2.23, 7.98)	<0.001
5 to <10 years	17	38	4.22 (2.00, 8.88)	5.30 (2.38, 11.81)	<0.001
≥10 years	4	13	1.49 (0.35, 6.41)	1.80 (0.41, 7.94)	0.44

[a]Adjusted for age, birth country, marital status, education, income, and region.
[b]Additionally adjusted for prior history of psychiatric disorders (major depression, anxiety disorder, bipolar disorder, schizophrenia, AUD, DUD) at index date.
AUD = alcohol use disorder, CI = confidence interval, DUD = drug use disorders, HR = hazard ratio, PC = prostate cancer

Supplementary Table 4. Associations between PC diagnosis (1998-2017) and risk of DUD through 2018, after excluding men with any diagnosis of DUD before index date.

**Time after PC diagnosis**	**DUD, n**		**Adjusted HR (95% CI)[a]**	***P***
	**PC cases**	**Controls**		
**High-risk PC**				
Entire follow-up period	345	2,043	1.86 (1.60, 2.15)	<0.001
<3 months	20	168	3.86 (1.97, 7.57)	<0.001
3 to <12 months	43	276	1.39 (0.90, 2.15)	0.14
1 to <2 years	51	319	1.95 (1.31, 2.90)	0.001
2 to <5 years	100	628	1.80 (1.38, 2.36)	<0.001
5 to <10 years	90	478	1.80 (1.35, 2.40)	<0.001
≥10 years	41	174	2.10 (1.36, 3.26)	0.001
**High-risk PC (2005-2017)[b]**				
ADT only	112	--	2.50 (1.89, 3.30)	<0.001
Radiation	59	--	1.75 (1.22, 2.51)	0.002
Radical prostatectomy	6	--	0.65 (0.20, 2.11)	0.48
Radical prostatectomy and radiation	7	--	0.70 (0.23, 2.15)	0.54
**Low- or intermediate-risk PC**				
Entire follow-up period	474	4,325	1.04 (0.92, 1.17)	0.56
<3 months	13	316	0.55 (0.25, 1.25)	0.15
3 to <12 months	41	468	0.72 (0.47, 1.09)	0.12
1 to <2 years	55	573	0.97 (0.68, 1.38)	0.87
2 to <5 years	136	1,282	1.08 (0.87, 1.35)	0.49
5 to <10 years	151	1,203	1.12 (0.90, 1.39)	0.30
≥10 years	78	483	1.15 (0.84, 1.58)	0.38
**Low- or intermediate-risk PC (2005-2017)[b]**				
Deferred treatment	169	--	0.97 (0.80, 1.19)	0.80
ADT only	48	--	1.94 (1.25, 3.01)	0.003
Radiation	49	--	0.87 (0.60, 1.26)	0.46
Radical prostatectomy	35	--	0.89 (0.58, 1.38)	0.60
Radical prostatectomy and radiation	7	--	1.35 (0.56, 3.22)	0.51

[a]Adjusted for age, birth country, marital status, education, income, region, and prior history of psychiatric disorders (major depression, anxiety disorder, bipolar disorder, schizophrenia, AUD) at index date.

AUD = alcohol use disorder, CI = confidence interval, DUD = drug use disorders, HR = hazard ratio, PC = prostate cancer

Supplementary Table 5. Associations between PC diagnosis (1998-2017) and risk of opioid use disorder through 2018, Sweden.

**Time after PC diagnosis**	**Opioid use disorder, n**		**Adjusted HR (95% CI)[a]**	***P***
	**PC cases**	**Controls**		
**High-risk PC**				
Entire follow-up period	182	1,515	1.69 (1.39, 2.06)	<0.001
<3 months	12	198	1.16 (0.38, 3.48)	0.80
3 to <12 months	23	140	1.44 (0.81, 2.54)	0.21
1 to <2 years	30	151	1.27 (0.78, 2.06)	0.34
2 to <5 years	47	355	1.12 (0.78, 1.61)	0.54
5 to <10 years	48	412	2.03 (1.39, 2.97)	<0.001
≥10 years	22	259	3.07 (1.61, 5.84)	0.001
**High-risk PC (2005-2017)[b]**				
ADT only	65	--	2.22 (1.55, 3.17)	<0.001
Radiation	38	--	2.05 (1.25, 3.34)	0.004
**Low- or intermediate-risk PC**				
Entire follow-up period	198	2,199	1.02 (0.84, 1.25)	0.85
<3 months	11	375	1.21 (0.37, 3.98)	0.75
3 to <12 months	27	253	0.75 (0.39, 1.46)	0.40
1 to <2 years	20	225	0.74 (0.40, 1.40)	0.36
2 to <5 years	49	498	0.85 (0.58, 1.23)	0.38
5 to <10 years	51	543	0.99 (0.69, 1.42)	0.96
≥10 years	30	305	1.43 (0.88, 2.30)	0.15
**Low- or intermediate-risk PC (2005-2017)[b]**				
Deferred treatment	67	--	0.89 (0.62, 1.27)	0.52
ADT only	12	--	1.34 (0.61, 2.90)	0.47
Radiation	21	--	0.65 (0.35, 1.24)	0.19

[a]Adjusted for age, birth country, marital status, education, income, region, and prior history of psychiatric disorders (major depression, anxiety disorder, bipolar disorder, schizophrenia, AUD, DUD) at index date.
[b]Subanalysis based on treatment data available during 2005-2017.
ADT = androgen deprivation therapy, AUD = alcohol use disorder, CI = confidence interval, DUD = drug use disorders, HR = hazard ratio, PC = prostate cancer

Supplementary Table 6. Associations between PC diagnosis (1998-2017) and risk of sedative/hypnotic use disorder through 2018, Sweden.

**Time after PC diagnosis**	**Sedative/hypnotic use disorder, n**		**Adjusted HR (95% CI)[a]**	***P***
	**PC cases**	**Controls**		
**High-risk PC**				
Entire follow-up period	98	1,183	1.20 (0.90, 1.59)	0.21
<3 months	15	198	3.19 (1.24, 8.22)	0.02
3 to <12 months	9	117	0.47 (0.19, 1.19)	0.11
1 to <2 years	14	101	1.13 (0.54, 2.39)	0.75
2 to <5 years	26	287	1.20 (0.72, 2.00)	0.48
5 to <10 years	27	277	0.88 (0.51, 1.53)	0.66
≥10 years	7	203	0.85 (0.29, 2.45)	0.76
**High-risk PC (2005-2017)[b]**				
ADT only	34	--	1.95 (1.14, 3.33)	0.02
Radiation	13	--	0.56 (0.23, 1.36)	0.20
**Low- or intermediate-risk PC**				
Entire follow-up period	187	1,879	1.08 (0.85, 1.38)	0.54
<3 months	14	356	1.13 (0.26, 4.98)	0.87
3 to <12 months	27	207	0.41 (0.17, 1.01)	0.05
1 to <2 years	27	231	0.90 (0.43, 1.91)	0.79
2 to <5 years	52	420	1.25 (0.82, 1.92)	0.30
5 to <10 years	49	436	1.19 (0.77, 1.83)	0.43
≥10 years	18	229	0.95 (0.45, 2.04)	0.90
**Low- or intermediate-risk PC (2005-2017)[b]**				
Deferred treatment	87	--	1.11 (0.72, 1.70)	0.64
ADT only	17	--	3.43 (1.37, 8.56)	0.008
Radiation	17	--	1.22 (0.56, 2.65)	0.62

[a]Adjusted for age, birth country, marital status, education, income, region, and prior history of psychiatric disorders (major depression, anxiety disorder, bipolar disorder, schizophrenia, AUD, DUD) at index date.
[b]Subanalysis based on treatment data available during 2005-2017.
ADT = androgen deprivation therapy, AUD = alcohol use disorder, CI = confidence interval, DUD = drug use disorders, HR = hazard ratio, PC = prostate cancer

Supplementary Table 7. Associations between PC diagnosis (1998-2017) and risk of AUD through 2018, stratified by age at index date.

Age at index date	AUD, n		Reduced model[a]	Full model[b]	
	PC cases	Controls	HR (95% CI)	HR (95% CI)	*P*
**High-risk PC**					
<55 years	40	394	1.80 (1.35, 2.41)	1.66 (1.18, 2.34)	0.004
55-64 years	327	3,574	1.60 (1.42, 1.79)	1.53 (1.33, 1.75)	<0.001
65-74 years	544	7,452	1.39 (1.26, 1.54)	1.39 (1.22, 1.57)	<0.001
75-84 years	197	4,119	1.23 (1.03, 1.46)	1.31 (1.06, 1.64)	0.02
≥85 years	20	546	1.23 (0.65, 2.34)	1.49 (0.69, 3.22)	0.31
**Low- or intermediate-risk PC**					
<55 years	191	2,130	1.46 (1.27, 1.68)	1.67 (1.41, 1.98)	<0.001
55-64 years	1,080	12,753	1.25 (1.17, 1.34)	1.42 (1.31, 1.53)	<0.001
65-74 years	1,123	15,810	1.10 (1.02, 1.19)	1.28 (1.17, 1.41)	<0.001
75-84 years	153	2,939	1.01 (0.81, 1.25)	1.22 (0.93, 1.61)	0.16
≥85 years	5	104	0.15 (0.02, 1.36)	0.23 (0.01, 3.59)	0.29

[a]Adjusted for age, birth country, marital status, education, income, and region.

[b]Additionally adjusted for prior history of psychiatric disorders (major depression, anxiety disorder, bipolar disorder, schizophrenia, AUD, DUD) at index date.

AUD = alcohol use disorder, CI = confidence interval, DUD = drug use disorders, HR = hazard ratio, PC = prostate cancer

Supplementary Table 8. Associations between PC diagnosis (1998-2017) and risk of DUD through 2018, stratified by age at index date.

**Age at index date**	**DUD, n**		**Reduced model[a]**	**Full model[b]**	
	**PC cases**	**Controls**	**HR (95% CI)**	**HR (95% CI)**	***P***
**High-risk PC**					
<55 years	15	130	1.32 (0.80, 2.15)	1.09 (0.52, 2.25)	0.83
55-64 years	74	550	1.61 (1.25, 2.07)	1.96 (1.45, 2.65)	<0.001
65-74 years	81	769	1.84 (1.48, 2.29)	2.25 (1.74, 2.91)	<0.001
75-84 years	73	593	1.78 (1.39, 2.29)	1.71 (1.31, 2.24)	<0.001
≥85 years	19	143	2.01 (1.12, 3.60)	1.98 (1.06, 3.71)	0.03
**Low- or intermediate-risk PC**					
<55 years	53	770	0.97 (0.75, 1.27)	1.16 (0.83, 1.64)	0.38
55-64 years	162	2,109	0.89 (0.76, 1.04)	1.02 (0.84, 1.25)	0.81
65-74 years	163	1,807	1.16 (0.98, 1.38)	1.24 (1.01, 1.52)	0.04
75-84 years	40	460	1.51 (1.09, 2.08)	1.69 (1.19, 2.41)	0.003
≥85 years	9	36	2.81 (1.11, 7.12)	4.03 (1.50, 10.82)	0.006

[a]Adjusted for age, birth country, marital status, education, income, and region.
[b]Additionally adjusted for prior history of psychiatric disorders (major depression, anxiety disorder, bipolar disorder, schizophrenia, AUD, DUD) at index date.
AUD = alcohol use disorder, CI = confidence interval, DUD = drug use disorders, HR = hazard ratio, PC = prostate cancer

Supplementary Table 9. Associations between high-risk PC (1998-2017), stratified by year of diagnosis, and risk of AUD or DUD within the next 2 years.

Calendar year of PC diagnosis	No. with outcome		Reduced model[a]	Full model[b]	
	PC cases	Controls	HR (95% CI)	HR (95% CI)	*P*
**AUD**					
1998-2004	276	2,962	1.20 (1.03, 1.40)	1.13 (0.93, 1.36)	0.21
2005-2009	486	5,987	1.75 (1.53, 1.99)	1.81 (1.55, 2.11)	<0.001
2010-2017	701	9,671	1.56 (1.40, 1.74)	1.48 (1.29, 1.70)	<0.001
**DUD**					
1998-2004	12	72	2.02 (1.29, 3.19)	2.32 (1.37, 3.92)	0.002
2005-2009	12	122	1.48 (0.98, 2.24)	1.33 (0.78, 2.27)	0.30
2010-2017	38	317	2.00 (1.50, 2.66)	2.27 (1.56, 3.31)	<0.001

[a]Adjusted for age, birth country, marital status, education, income, and region.
[b]Additionally adjusted for prior history of psychiatric disorders (major depression, anxiety disorder, bipolar disorder, schizophrenia, AUD, DUD) at index date.
AUD = alcohol use disorder, CI = confidence interval, DUD = drug use disorders, HR = hazard ratio, PC = prostate cancer
